# Diagnostic Modalities in Detecting Cardiovascular Complications of Thalassemia

**DOI:** 10.31083/j.rcm2308267

**Published:** 2022-07-22

**Authors:** Pandji I. Fianza, Alvinsyah A. Pramono, Mohammad Ghozali, Teddy A. Sihite, Djatnika Setiabudi, Mas R. A. A. Syamsunarno, Suthat Fucharoen, Ramdan Panigoro

**Affiliations:** ^1^Department of Internal Medicine, Division of Hematology and Medical Oncology, Faculty of Medicine, Universitas Padjadjaran/Hasan Sadikin General Hospital, 40161 Bandung, West Java, Indonesia; ^2^Research Center of Medical Genetics, Faculty of Medicine, Universitas Padjadjaran, 40161 Bandung, West Java, Indonesia; ^3^Doctoral Study Program, Faculty of Medicine, Universitas Padjadjaran, 40161 Bandung, West Java, Indonesia; ^4^Department of Biomedical Sciences, Faculty of Medicine, Universitas Padjadjaran, 40161 Bandung, West Java, Indonesia; ^5^Department of Cardiology and Vascular Medicine, Faculty of Medicine, Universitas Padjadjaran/Hasan Sadikin General Hospital, 40161 Bandung, West Java, Indonesia; ^6^Department of Child Health, Faculty of Medicine, Universitas Padjadjaran/Hasan Sadikin General Hospital, 40161 Bandung, West Java, Indonesia; ^7^Thalassemia Research Center, Institute of Molecular Biosciences, Mahidol University, 10400 Nakhonpathom, Thailand

**Keywords:** cardiac siderosis, diagnostic modalities, thalassemia major

## Abstract

Thalassemia major is the most common monogenetic disorder worldwide, manifested as chronic hemolytic anemia. This condition leads to the need for chronic blood transfusion to be monitored for an iron overload that may be stored in several tissues and organs, including cardiomyocytes, that might cause a broad spectrum of cardiac iron toxicities such as heart failure conduction delays, myocarditis, and arrhythmias. Non-invasive imaging modalities have their benefits and limitations. Each modality complements and generates a comprehensive diagnostic and monitoring of cardiac siderosis in thalassemia major patients.

## 1. Introduction

Thalassemia major (TM), also known as transfusion-dependent thalassemia (TDT), is the most common disorder that manifests as chronic hemolytic anemia affecting a patient for a lifetime [1, 2]. TDT requires regular blood transfusion to prolong and improve the quality of life [1, 2]. However, patients with TDT who receive chronic blood transfusion should be monitored for potential iron overload. Iron overload may occur in several tissues and organs, including the heart, and might cause a vast spectrum of cardiac iron toxicities such as heart failure, conduction delays, myocarditis, and arrhythmias [3, 4, 5, 6, 7]. Unlike common myocarditis due to infection, cardiac toxicities alone could be the etiology of myocarditis in TDT patients [8, 9]. To date, cardiac complications are known as the leading cause of death in TDT [7, 10, 11].

Managing TDT and its complications, as well as its comorbidities, has been an enormous challenge for decades. Since its introduction in the 1970s, iron chelator agents have been going through numerous trials to optimize their performance in chelating labile iron, thus preventing iron toxicities in TDT patients [7]. Thus far, combined chelating agents and heart failure management have been recognized as management strategies of TDT alongside regular lifelong blood transfusion. Clinical trials on iron chelators and their combination regimens have been well described in a review article by Chapin J *et al*. [12]. However, a recently published study curating national thalassemia registry data has indicated that iron chelator agents are not evenly accessible for all TDT patients [12]. Younger TDT patients are likely to be prescribed combination iron chelators, yet they exhibited significantly higher mean serum ferritin concentration during a 12-month interval period of observation. Meanwhile, older patients, mainly prescribed deferasirox (DFX), have demonstrated lower mean cardiac T2* that correlated with higher cardiac levels than younger patients [12].

Despite the adequate regimen to prevent iron toxicity, it is also essential to regularly monitor and screen for cardiac progressivity to guide the clinical management and decision to improve the quality of life of TDT patients. Patients with TDT must undergo anatomical structure screening and heart physiological function screening to prevent cardiac iron toxicities that lead to deaths of TDT patients. Hence, this article attempts to summarize available studies on the pathophysiological process of cardiac complications and diagnostic tools that physicians can utilize to monitor cardiac complications in TDT patients. In this review, available evidence elaborating the use of non-invasive imaging examination to diagnose cardiac complications in Thalassemia is included. Joanna Briggs Institute critical appraisal tool is utilized to ensure the included studies are of the highest quality.

## 2. Pathophysiology of Cardiac Complications in Thalassemia

Cardiac compensation is a frequent mechanism observed in chronic anemia caused by TDT to ensure sufficient oxygenation to peripheral tissues and organs [4]. Chronic anemia in TDT patients is known to impair the cardiac sympathetic and parasympathetic signals, creating a spectrum of heart rate and rhythm abnormalities. The study by Kumfu *et al*. [13], which reviewed studies from animal-to-human observations, described that autonomous cardiac impairment creates a spectrum of heart variabilities, including increased cardiac output, stroke volume, heart rate, or combination of these variabilities. It has been observed that both the average and minimum heart rates of thalassemia patients are significantly higher than non-thalassemia patients [14]. This mechanism is in line with several translational studies observing cardiac physiology in β-thalassemia mice models [13]. The article has well described recorded changes in β-thalassemia mice models, including increased heart weight, cardiac iron concentration, stroke volume, and cardiac output [13].

Increased cardiac load in TDT is linked to increased blood pressure achieved by decreasing total peripheral resistance to stabilize blood pressure, dilate peripheral arteries, widen pulse pressure, and significantly reduce diastolic pressure [4].

Higher concentrations of hemoglobin F (HbF) in TDT patients and lower concentrations of 2,3-biphosphoglycerate in transfused blood aggravate tissue hypoxia. Tissue hypoxia is known to expand bone marrow tissue to increase the production of hematopoietic stem cells and tissues. High turnover of red blood cells causes enlargement of the spleen and increases intrasplenic circulation. Both mechanisms generally increase cardiac output in TDT patients. In addition, advanced liver damage following chronic transfusion might contribute to increased cardiac output in TDT [7].

Transferrin is a blood plasma protein responsible for binding and circulating ferrous iron in the body. Chronic blood transfusions approximately load 5.8–11.6 grams of iron in a 50-kilogram TDT patient annually, causing physiologically available transferrin to be highly saturated [15]. A high concentration of unbound labile iron in the circulation enters cardiomyocytes through voltage-dependent L-type calcium channels. In the cardiomyocytes, iron is stored in ferritin, hemosiderin, and labile iron [4]. Labile iron, also known as non-transferrin bound iron (NTBI), is the most cardiotoxic form of iron stored in the heart. NTBI is buffered by ferritin in the cardiomyocytes as a ferritin-iron complex in the liposome-derived cellular body for long-term storage to prevent oxidative damage in the heart [4, 7]. Therefore, it explains subclinical cardiac siderosis states that may last longer in TDT patients.

**Fig. 1.**
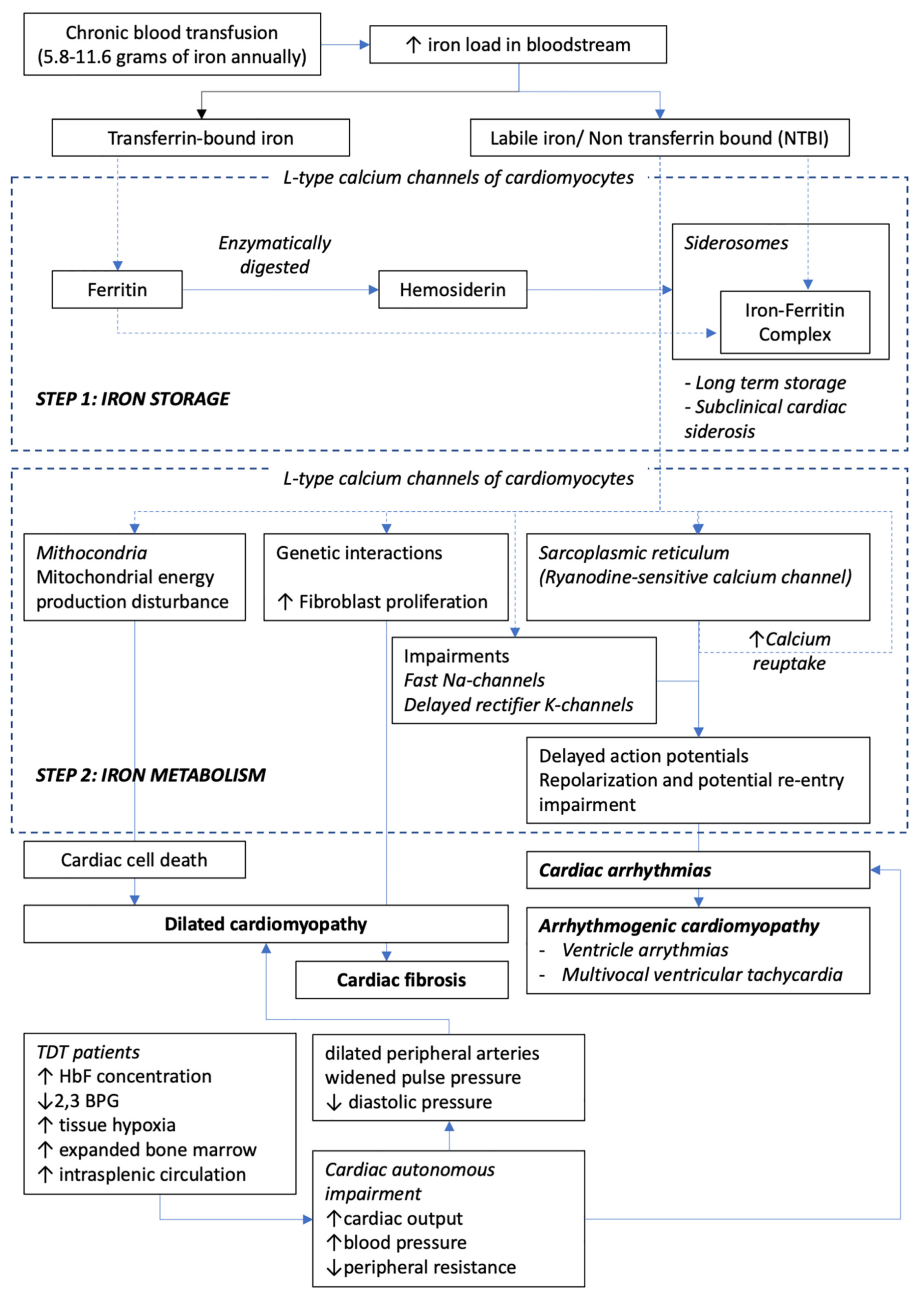
**Pathomechanism of cardiac iron toxicity in TDT patients**. Chronically transfused TDT patients gain 5.8–11.6 grams of iron annually. Labile iron is stored as non-transferrin bound iron (NTBI) in siderosomes, creating an iron-ferritin complex that may be stable for years. On the other hand, NTBI interacts with cardiomyocytes creating impairments that lead to cardiomyopathy and events of arrhythmias, causing the heart to be the most targeted organ in thalassemia. Na, sodium; K, potassium; HbF, Hemoglobin F; 2,3 BPG, 2,3 biphosphoglycerate.

Interactions between labile iron and cardiomyocytes induce several conditions in the cardiac muscle cells, including impaired mitochondrial metabolism, genetic modulation, and ion channel interactions. Mitochondrial uptake of labile iron causes metabolic disturbance of mitochondrial energy production, leading to cardiac cell death and dilated cardiomyopathy [4]. On the other hand, increased iron in cardiomyocytes typically cause genetic interactions that induce fibroblast proliferation in the heart, owing to cardiac fibrosis [4]. Labile iron also interacts with the sarcoplasmic reticulum through the ryanodine-sensitive calcium channel. Increased concentration of labile iron is associated with calcium reuptake modulation through the channel, causing arrhythmias and arrhythmogenic cardiomyopathy [4]. Impairment of the fast sodium and delayed-rectifier potassium channels caused by intracellular labile iron in the myocytes are known to disturb the cardiac conduction process that may cause several clinical signs, including widened QRS complex of the ECG (electrocardiography), delayed-action potential distribution in the myocardium, as well as repolarization and possible re-entry impairment such as ventricle arrhythmias and multivocal ventricular tachycardia (*torsade de points*) (Fig. 1) [4].

## 3. Diagnostic Modalities for Cardiovascular Manifestation in Thalassemia 

Diagnostic modalities in detecting cardiovascular diseases, especially in TDT, have been well developed. Since cardiac complications are the leading cause of death in thalassemia patients, it is crucial to thoroughly understand the diagnostic tools required for structural and physiological function screening of the heart.

### 3.1 Electrocardiography 

Electrocardiography (ECG) is one of the most basic and routinely conducted tests in daily clinical, hospital, and emergency settings. ECG is commonly used to detect anatomical and heart rhythm changes based on the heart’s electrical activity [16, 17]. While ECG is not the gold standard tool in diagnosing cardiac iron accumulation, in the absence of cardiac T2* magnetic resonance imaging (MRI), ECG offers a highly accessible and cost-effective tool to assess cardiac iron toxicity in thalassemia patients [18].

Various ECG abnormalities, including tachycardia, T-wave inversion, left ventricular hypertrophy, and supraventricular extrasystole, are commonly discovered in patients with ferritin serum exceeding 2500 ng/mL [19]. QRS complex duration and corrected QT are significantly prolonged and correlated with serum ferritin level. In addition, corrected QT is inversely correlated with the T2* value in pediatric and adolescent TDT patients [18, 20, 21]. Paroxysmal atrial fibrillation, S1Q3 pattern, and right QRS axis deviation are also observed in TDT patients developing signs and symptoms of heart failure [22].

Another study recorded low limb voltage, flattening p wave, and premature atrial complex that appeared subclinical [21]. Recent studies also indicated that recorded ECG of older thalassemia patients demonstrated significantly prolonged PR interval, filtered QRS duration, low-amplitude signal duration, and shortened root mean square QRS size at final 40 msec that are increasingly more severe over time [23, 24]. Both ECG and signal-averaged ECG showed significant differences in cardiac electrical impulse correlated with ferritin levels, suggesting iron toxicities as one of the postulated underlying causes of the ECG abnormalities [24]. Since cardiac iron deposition creates a toxic-oxidant environment for the cardiomyocytes, recent studies have indicated that the treatment of N-acetylcysteine, an antioxidant, to TDT patients demonstrated improvement in their Holter ECG results [25]. Overall, ECG may serve as an option to monitor early changes in cardiac iron toxicities. It is widely available and able to provide a lot of information that helps physicians prevent fatality among TDT patients.

### 3.2 Echocardiography

Echocardiography (Echo) is a non-invasive examination that utilizes ultrasound to capture the anatomical structure and functions of the heart that a cardiologist commonly performs [26]. Echocardiography parameters are widely used to determine structural changes in the heart that are assumed to correlate with hemodynamic and physiological changes in patients [27].

Several studies demonstrated that thalassemia patients, especially those with cardiac iron toxicity, might indicate several changes in their echocardiographic parameters, including significantly decreased atrial filling velocity (A) duration, higher left atrial deformation, higher early ventricular filling velocity (E), higher pulmonary vein diastolic filling velocity (PVD), a lower ratio of pulmonary vein systolic/diastolic filling velocity (PVS/PVD), higher pulmonary vein atrial reversal filling velocity (PVAR) duration, and lower septal early diastolic myocardial velocity (E’) than non-thalassemia patients [23, 28, 29]. In clinical terms, changes in echocardiographic parameters could present atrial fibrillation and left ventricular dysfunction that worsens patients’ hemodynamics [23].

Thalassemia patients were also found to have larger left ventricular mass, and larger left ventricular systolic and diastolic diameters, suggesting left ventricle hypertrophy [30]. These findings were also associated with mitral and tricuspid valve insufficiency and positively correlated with NT pro-BNP. The cardiac muscle releases this biomarker due to the stretching of the cardiac myocytes [30]. Echo presents a set of dynamic measurements of heart anatomical and physiological structure that assist TDT patients in monitoring their cardiac conditions after chronic transfusion.

In addition, it has been demonstrated that a higher level of ferritin is significantly correlated with a higher grade of diastolic dysfunction [28]. As diastolic dysfunction precedes any systolic dysfunction in many cardiac diseases, it is essential to monitor the progressivity of cardiac structure that might be interfered with in TDT patients, including left atrium and left ventricular strain, strain rate, mitral inflow, and annular velocities. Echo measurement of both TDT and NTDT (Non-Tranfusion Dependent Thalassemia) patients revealed LV end-diastolic diameter (LVEDD), septal E and A velocities, and size of LA (Left Atrium) area (both systole and diastole) to be significantly higher than average [31, 32].

Pulmonary hypertension was one of the complications that might be observed in TDT patients’ echocardiogram. Tricuspid regurgitant jet velocity (TRJV) of more than 2.8 m/s with exertional dyspnea and absence of left heart failure indicate the presence of pulmonary hypertension. TRJV >2.8 m/s is significantly associated with a higher reticulocyte and lactate dehydrogenase level in TDT patients [33]. According to the abovementioned criteria, it was discovered that 4.5% of chronically transfused β-thalassemia patients experienced pulmonary hypertension [34, 35].

To date, more advanced echocardiography methods such as 2D or 3D speckle tracing could reflect early abnormalities without any clinical relevance. In a study by Rozwadowska *et al*. [36], 2D speckle tracing is sensitive to detect subclinical systolic dysfunction in patients with TDT. However, the correlations between strain imaging parameters and T2* values are lacking. Since this technique is still in development, the measurement technique of 2D or 3D speckle tracking should improve their intervendor reliability. In addition, more studies are required to determine the relevance of early abnormalities and their correlation to treatment and prognosis [36, 37].

Echo provides a more sensitive and accurate diagnosis in monitoring cardiac progressivity in TDT patients. In Indonesia, echocardiography is mostly accessible in referral hospitals. However, basic measurement techniques which should be performed in any clinical conditions in cardiovascular assessment should also be fully comprehended by cardiologists in remote areas [38]. When a cardiologist performs an echocardiography, the diagnosis is user-dependent. In addition, Echo is not able to quantify iron stored in the cardiac muscles. Therefore, it is crucial to double-check clinical signs, Echo, and iron concentration through a more sensitive measurement.

### 3.3 Magnetic Resonance Imaging

Magnetic resonance imaging (MRI) is a non-invasive, susceptible, and specific measurement free from radiation, making it safer for both patients and radiologists [16]. Even though it is not as economical as ECG and echocardiography, MRI offers a more delicate and sophisticated technology that produces a more reliable, reproducible, and standardized result for a better diagnostic process [16, 39].

MRI images for iron quantification are created from perceived desynchronized water proton by the MRI scanner through radiofrequency pulse (rf), spin echo, or gradient echo formation [40]. A darker resultant image is produced from a longer echo time (TE) [40]. Iron-mediated darkening may be recognized by a half-time constant, known as T2 or T2*. Both T2 and T2* are standard terms observed if spin-echo and gradient-echo are used as quantifying methods [40]. T2 and T2* measurement is more stable in iron-loaded tissues and clinically relevant with other parameters measured [40]. The drawback of the gradient-echo technique is that the “minimum echo time” is in fact a problem for the liver, not the heart. In reality, it is almost impossible to have patients with a cardiac T2* <2 ms. If the minimum echo time of T2* is too long, most of the MRI signals will have irreversibly disappeared when it is reached. Therefore, captured images will present lightly iron-loaded tissues and inaccurately reflect the absolute iron deposition [40, 41].

Cardiac T2* MRI allows non-invasive quantifications of myocardial iron burden in all patients, including TDT. T2* values of less than 10 ms, 10–14 ms, and 14–20 ms are considered severe, moderate, and acceptable cardiac T2* respectively [42, 43]. Examination of cardiac T2* has been validated in human studies to investigate cardiac iron toxicity in human patients. Numerous studies demonstrated that cardiac T2* has a significant correlation with serum ferritin levels in TDT patients [42, 44]. In TDT patients, cardiac T2* has also a substantial correlation with various echocardiography parameters such as mitral annulus systolic velocity (S’), myocardial performance index (MPI), and Global longitudinal strain (GVLS) [42, 43, 45]. Therefore, in conditions where MRI cannot be conducted, an evaluation of cardiac iron overload in TDT patients using serum ferritin and echocardiography should at least be conducted to monitor cardiac complication in TDT patients [44]. Translating BELIEVE trial to clinical practice, a BELIEVE trial examined TDT patients treated with Luspatercept that improved quality of life and correlated with serum ferritin and cardiac T2*. A ten-year-long cohort of TDT patients demonstrated that a high-risk cardiac T2* value is associated with decreased survival rate in the long term [46]. Although various studies describe a correlation between cardiac T2* and serum ferritin with their primary end point, it is noted that numerous studies found no correlations of cardiac T2* and serum ferritin in TDT patients [47]. Hence, cardiac T2* alone cannot be replaced with any other measurements to measure cardiac complications in TDT patients.

Another study suggested that cardiac iron toxicity might be observed in childhood, as early as the first five years of life in inadequate iron chelator therapy populations, indicating that CMR (cardiovascular magnetic resonance) should be utilized as early as possible to monitor TDT quantitatively patients [48]. A lower median T2* level is commonly discovered in children consuming desferrioxamine (DFO) and deferasirox (DFX) [34].

As an alternative to T2* parameters, segmental analysis of T1 is considered more sensitive than T2* to monitor cardiac complications in TDT patients [49]. To date, MRI is also the gold standard for quantifying biventricular volumes and functions that could also detect myocardial fibrosis [50, 51]. Besides iron overload, myocardial fibrosis (both focal and diffuse) is a frequent finding in TDT patients, associated with cardiovascular complications [50]. Therefore, cardiac T2* and T1 MRI are highly recommended to measure the iron concentration in cardiac muscles, regardless of the results of serum ferritin, ECG, and Echo measurement [52].

## 4. Conclusions

This article summarizes the cardiovascular manifestations and diagnostic modalities in thalassemia patients. ECG, Echo, and MRI have their benefits as well as limitations. Each modality complements each other and generates a comprehensive diagnostic and monitoring of cardiac siderosis in TDT patients.
